# An In Vitro Evaluation of Morinda citrifolia and Ocimum sanctum as Potential Storage Media to Maintain Cell Viability for Avulsed Teeth Using Collagenase Dispase Assay

**DOI:** 10.7759/cureus.36837

**Published:** 2023-03-29

**Authors:** Shweta Vijaykumar Sagare, Anand Patil, Pranav Patıl, R. Susheel Kumar, Sairam Gangishetti, Priya Ingale

**Affiliations:** 1 Department of Conservative Dentistry and Endodontics, Bharati Vidyapeeth (Deemed To Be University) Dental College and Hospital, Sangli, IND; 2 Department of Conservative Dentistry and Endodontics, KLE Vishwanath Katti Institute of Dental Sciences and Hospital, KLE Academy of Higher Education and Research, Belagavi, IND; 3 Department of Conservative Dentistry and Endodontics, Bharati Vidyapeeth (Demeed To Be University) Dental College and Hospital, Sangli, IND; 4 Department of Pedodontics and Preventive Dentistry, Panineeya Dental College and Hospital, Hyderabad, IND; 5 Department of Conservative Dentistry and Endodontics, Meghna Institute of Dental Sciences, Nizamabad, IND; 6 Department of Conservative Dentistry and Endodontics, Vastandada Patil Dental College and Hospital, Sangli, IND

**Keywords:** ocimum sanctum, morinda citrifolia, hbss, collagenase, avulsed teeth

## Abstract

Aim

This study aimed to evaluate the protective effects of *Ocimum sanctum *extract and *Morinda citrifolia *juice on human periodontal ligament (PDL) cells after the reimplantation of avulsed teeth using a collagenase-dispase test.

Materials and methods

Sixty-five human premolars, all of which would eventually need to be extracted, were split into three experimental groups: one treated with Hanks Balanced Salt Solution, another with Morinda citrifolia juice and Ocimum sanctum extract, and two control groups (positive and negative).* *There were 10 teeth in each control group and 15 teeth each were used in the experimental groups, with the first 30 minutes spent dry before being submerged in one of three experimental media for 45 minutes, followed by 30 minutes of treatment with collagenase and dispase II. The cells' vitality was measured by the trypsin dye exclusion technique. To determine how many PDL cells were still alive, An optical microscope and a hemocytometer were used. The data were analyzed using Kruskal-Wallis one-way ANOVA and Mann-Whitney U tests.

Results

The percentage of viable PDL cells was greatest in *Morinda citrifolia *juice (85.18%), followed by HBSS (84.3%), and finally by *Ocimum sanctum *extract (68.04%). There was no significant difference in the number of viable PDL cells in *Morinda citrifolia *juice and HBSS.

Conclusion

The results of this research suggest that *Morinda citrifolia *juice has potential as a storage medium and as an alternative to HBSS, within the study's constraints, considering its availability as well as economic feasibility.

## Introduction

Avulsion occurs when the tooth is entirely dislodged from its socket. Avulsions are the most common kind of traumatic injury to permanent dentition, occurring in 1% to 16% of cases [1]. Dental avulsion is a complex injury that damages several tissue compartments, including the cementum, alveolar bone, gingival soft tissue, periodontal ligament, and dental pulp [1].

Avulsion injuries often result in some degree of attachment apparatus damage. Nonetheless, it is essential to maintain the health of the periodontal ligament (PDL) attached to the avulsed tooth. Successful replantation relies in large part on PDL cells because of their profound effect on resorption. The amount of time the tooth spends dry outside of the socket after being avulsed is a major determinant in deciding the tooth's prognosis. In order to protect PDL cell viability, promote healing, and reduce root resorption, the tooth should be replanted into the socket as soon as possible. There is less than a 50% likelihood of periodontal repair if replantation is delayed by even 8 minutes [2]. The objective is, then, to keep the root surface PDL cells from being destroyed and to keep cellular metabolism running normally.

The survival of PDL components and dental pulp tissue in different interim transport media for avulsed teeth until the time of replantation has been the subject of a number of studies. It might be difficult and costly to acquire many of these formats [3].

The American Association of Endodontists (AAE) recommends storing avulsed teeth in Hanks Balanced Salt Solution (HBSS) because it may maintain the viability of the majority of PDL cells for extended periods of time. Osmolality-wise, HBSS is just right for supporting the development of cell type D: it has a neutral pH and is non-toxic. Nevertheless, widespread access to HBSS is lacking, limiting its use [4]. As a corollary, none of the prevalent media are perfect, and the quest for a media that can transcend these limits continues.

Tulsi, or "Holy Basil" in English, is the Hindi name for the plant *Ocimum sanctum *Linn., which has a long history of usage as a folk remedy for a wide range of medical issues in Indian households. Tulsi has anti-stress, immunoregulatory, anti-carcinogenic, antioxidant, and anti-microbial properties. It is rich in flavonoids, which are responsible for its antioxidant properties that aid in better collagenation, thereby leading to rapid wound healing [5]. Phytochemical constituents of Noni leaves are rich in flavonoids, which are shown to promote collagen formation, osteogenic differentiation, and matrix mineralization. This may help as an osteoinductive agent for bone and periodontal tissue regeneration [6, 7].

Based on the wound healing properties of *Morinda citrifolia *and *Ocimum sanctum*, in theory, they might be utilized as a medium to keep PDL cells from dying off in avulsed teeth. No studies using *Morinda citrifolia *juice or *Ocimum sanctum *extract for this purpose have been published as of yet. Therefore, the purpose of this in vitro study was to evaluate the efficacy of *Morinda citrifolia j*uice and *Ocimum sanctum *extract as storage media to maintain PDL cell viability of avulsed teeth using the collagenase -dispase assay.

## Materials and methods

Ethical approval was obtained from KLE Vishwanath Katti Institute of Dental Sciences​​​, Belagavi, Karnataka ethical committee with number IEC/2020/115. Sixty-five permanent human premolar teeth extracted for orthodontic purposes were collected to obtain periodontal fibroblast cell lines.

The inclusion criterion was permanent human premolar teeth extracted for orthodontic purposes. Teeth were obtained from participants with closed apices and without signs of caries or periodontal pathology. Extensively decayed teeth and teeth removed from individuals with periodontitis were not considered. The dental surgeon who extracted the teeth did so using less invasive procedures. As teeth were extracted, the crowns were held coronally in forceps while the coronal 3 mm of the PDL was scraped with a curette to remove any potentially injured cells.

Preparation of *Ocimum sanctum *extract

*Ocimum sanctum *leaves were picked fresh, cleaned twice with distilled water, and then air-dried for three to four days in the shade. To kill any bacteria that could be hiding in the dried leaves, they were subjected to UV light. The dried leaves, totaling 20 g, were chopped into bits before being added to 100 ml of double-distilled water and brought to a boil for 5 minutes. The water was cooled, filtered using No. 1.7 Whatman filter paper, and placed in the fridge for later use.

MTT (3-(4,5-Dimethylthiazol-2-yl)-2,5 diphenyltetrazolium bromide) assay

The concentration of the media (*Morinda citrifolia *and *Ocimum sanctum*) employed in the research was determined using an MTT test. After 24 hours of incubation at 37 degrees Celsius with 5% CO2, 2105 cells were sown in 96-well microtitre plates. Both the *Morinda citrifoila *and *Ocimum sanctum *test mediums were diluted to 10%, 5%, 2.5%, and 1.25% concentrations. The samples were prepared separately for the test and control groups and added in duplicate to the wells. A positive control consisted of untreated cells. During 24 hours, the plates were stored at 37 degrees Celsius, 5% CO2, and 50% humidity. After incubation for 4 hours at 37 degrees Celcius in the dark, 20 ul of MTT solution was added to each well. The MTT reagent and the culture media were disposed of with great care after incubation. To get rid of the precipitated formazan crystals, 200 µl of solvent (dimethyl sulfoxide) was poured into each well. The microtiter plates were agitated for 5 minutes at room temperature before being examined in a microplate reader set to 630 nm. The proportion of living cells was calculated using the given formula [6].

Cell Viability = (Optical density of Test compound / Optical density of Control) x 100

When the mean concentrations of *Morinda citrifolia *and *Ocimum sanctum *at 10%, 5%, 2.5%, and 1.25% were compared for PDL cell viability, 5% Noni (96.87%) and 5% Tulsi (93.18%) had the highest mean viable PDL cells. Therefore, these concentrations were used. The teeth of the experimental group were divided into three subgroups (n = 15) and allowed to air dry for 30 minutes before being immersed in water for 45 minutes. In Hank's neutral salt formula (HBSS), *Morinda citrifolia *juice (NONI), and *Ocimum sanctum *extract (TULSI) make up Groups I, II, and III which were the experimental groups, respectively. Ten control teeth (positive control) were given an instantaneous application of collagenase II and dispase II without being allowed to dry or being placed in a storage solution and ten control teeth (negative control) were collagenase II and dispase II treated after being bench dried in sterile containers for one hour with no further storage solution time [4].

**Figure 1:**
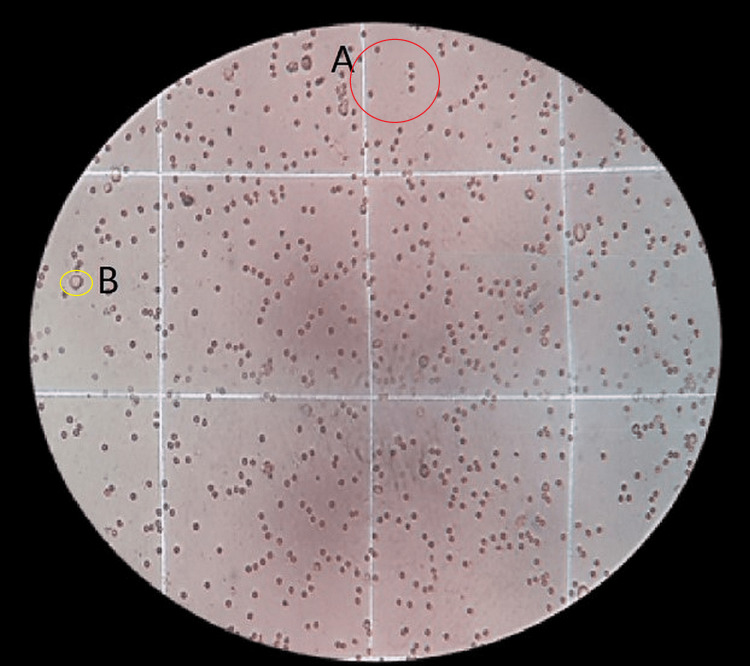
Microscope picture showing viable cells and non-viable cells A (red circle): Viable cells; B (yellow circle): Non-viable cells

Each experimental tooth was air dried, then soaked in phosphate-buffered saline for 30 minutes before being put in a 15 ml Falcon tube containing 2.5 ml of 0.2 mg/ml Collagenase II (HiMedia, Mumbai, India) and 2.4 ml of 0.2 mg/ml dispase grade II (Sigma-Aldrich, St. Louis, USA). Once the tubes were incubated for a while, they were topped up with 50 mL of fetal bovine serum (Gibco, Bengaluru, India). After a 4-minute centrifugation at 200 g, the concentration was calculated. In order to assess the condition of the periodontal ligament, the cells were stained with 0.4% trypan blue after the supernatant was drained. A trypan blue barrier keeps the dye outside of the cytoplasm in healthy cells. The extent of membrane damage present in the examined cells was gleaned from the percentage of survivors obtained using trypan blue dye exclusion. Using a hemocytometer connected to a microscope, the researchers determined what proportion of periodontal ligament cells were alive and what proportion were dead. Using this method, we were able to calculate the percentage of viable cells (Figure 1) using this formula [4]: The percentage of viable cells was obtained by using the mathematical equation: total number of viable cells/total number of cells (viable + non-viable) x 100.

Statistical analysis

One-way analysis of variance (ANOVA) and the Mann-Whitney U test were used for statistical analysis in SPSS 20.0 (IBM Corp., Armonk, USA); 5% was chosen as the level of significance with a 95% confidence level. The percentage of living cells in the five different groups was compared using Kruskal-Wallis ANOVA (HBSS, Noni, Tulsi, Positive, and Negative). Each of the five groups (HBSS, Noni, Tulsi, Positive, and Negative) had their % cell viability compared to one another using the Mann-Whitney U test.

## Results

The Kruskal-Wallis ANOVA test showed a significant difference between the groups (Table 1).

**Table 1:** Comparison of five groups (HBSS, Noni, Tulsi, Positive, and Negative) with respect to % viability using Kruskal-Wallis ANOVA HBSS: Hanks Balanced Salt Solution

Groups	N	Mean	SD	SE	Sum of ranks
HBSS	15	84.30	12.50	3.23	571.50
Noni	15	85.18	12.06	3.11	587.00
Tulsi	15	68.04	15.77	4.07	345.00
Positive Control	10	97.37	1.58	0.50	583.50
Negative control	10	12.75	13.07	4.13	58.00
Total	65	71.75	29.56	3.67	
H-value	45.5377				
p-value	0.00001				

**Figure 2:**
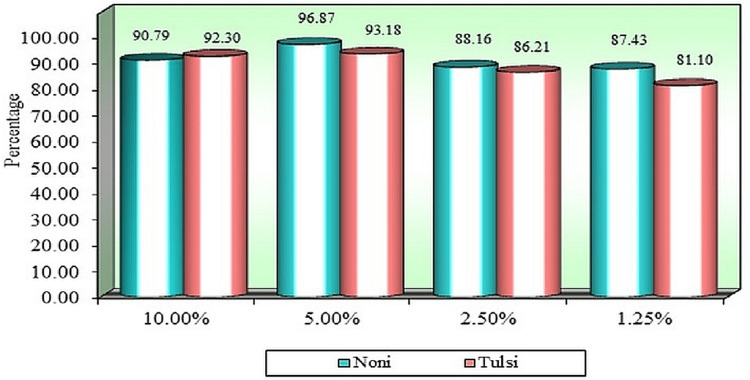
Comparison of various concentrations of Morinda citrifoila (Noni) and Ocimum sanctum (Tulsi) groups using MTT assay MTT: 3-(4,5-Dimethylthiazol-2-yl)-2,5 diphenyltetrazolium bromide

The median number of living cells was greatest in HBSS, next in 5% *Morinda citrifolia*, and finally in 5% *Ocimum sanctum*. No statistically significant difference (p > 0.05) was seen between HBSS and *Morinda citrifolia*. Pair-wise comparison of groups for viable cell number was performed by the Mann-Whitney U test procedure. However, HBSS and *Morinda citrifolia *showed a higher number of mean viable PDL cells compared to *Ocimum sanctum *(Figure 2).

## Discussion

It is recommended that teeth that have been knocked out due to trauma be replanted into their sockets as soon as possible [3] to avoid additional damage to the periodontal ligament cells due to desiccation. A blood clot forms between the alveolar and cemental parts of the PDL after reimplantation. Blood clots may organize as granulation tissue that regenerates under optimum circumstances for prompt replantation if the avulsed tooth is replanted with essential cemental PDL remnants. Yet, ex-articulated teeth are often replanted under adverse circumstances after being exposed to dry environments for an extended length of time. Due to cell necrosis, the number of functional PDL cells will decrease as a consequence [8]. Root resorption is a common complication of replanted teeth. PDL fibroblast death and collagen denaturation likely stimulate inflammatory resorption where bone can be replaced in the granulation tissue after tooth reimplantation. Another possible reason for the failure of reimplantation is bacterial contamination of the PDL. Bacterial products can present direct toxicity to PDL and can also enhance the inflammatory response after reimplantation. Avulsed teeth have a better chance of survival if they are replanted as soon as possible after being extracted and placed in a dry environment (additional oral dry time) [9]. Experts are debating the best way to store an avulsed tooth until it can be replanted to avoid the tooth's root being reabsorbed or replaced by inflammation. Therefore, healing after tooth avulsion and replantation depends on the extent of damage to the PDL. The longer the avulsed tooth is stored in a dry place, the worse the prognosis [10, 11].

Extra-alveolar dry time is less important than the storage medium's capacity to maintain cell viability in avoiding inflammatory and replacement root resorption [10]. For cells to remain alive and attach to the storage medium, it must be suitable for this purpose. Also, it has to be conveniently located close to the avulsion site. The medium's physiological osmolality and pH are critical for sustaining PDL cell viability. It has been hypothesized that between a pH of 6.6 and 7.8 and an osmolality of 230 to 400 mOsmol/kg, cells develop at their most rapid rates. Nevertheless, 290-330 mOsm [10] is the sweet spot for maximum development. Research on the best medium for preserving PDL cell viability and longevity has been conducted on several occasions. A number of different fluids, including saline, water, milk, saliva, HBSS, Gatorade, and contact lens solution, have been used to successfully maintain an avulsed tooth [4].

The American Association of Endodontists (AAE) recommends HBSS as a storage medium for avulsed teeth because of its proven success in promoting the growth of various cell types in laboratory settings. The pH of HBSS is 7 and its osmolality ranges from 270 to 290 mOsmol/kg. It contains sodium chloride, glucose, potassium chloride, sodium bicarbonate, and sodium phosphate, and calcium chloride, magnesium chloride, and magnesium sulfide are just some of the typical chemicals that might be used in this concoction. PDL cells' depleted biological components are maintained and restored by glucose, calcium, and magnesium. HBSS has a lengthy shelf life of 2 years. Save-A-Tooth (SmartPractice Dental, Phoenix, USA) is a commercially available option that has an interior mesh to accept the avulsed tooth, reducing the risk of cell damage during transit. Yet, its availability is restricted, especially in India [4, 11]*.*

The Noni or Indian mulberry tree, or more properly the *Morinda citrifolia *Linn. (Rubiaceae), is a tiny evergreen tree. It has been recommended to be used for many conditions, including arthritis, atherosclerosis, skin irritation, and wounds. The leaves of this plant are used topically on wounds to speed up their recovery. There is preliminary evidence from a few animal studies that Noni has anti-cancer, immune-boosting, and pain-relieving qualities [12]. In a recent study, Takashima et al. [13] showed that several of the novel chemicals they extracted from Noni leaves had therapeutic effects. Traditional therapeutic methods have included the application of a crude preparation of Noni leaf on wounds. According to a study done on streptozotocin-induced diabetic rats, ingesting the juice of the *Morinda citrifolia *tree has been shown to greatly enhance collagen production, which might speed up the recovery process. An ethanolic extract of Noni leaves was investigated for its wound-healing properties using excision and dead space wound models in rats by Nayak et al. [12]. It has been shown via scientific research that the majority of the granulation tissue is created during the proliferative phase, and that this tissue is composed of fibroblasts, collagen, edema, and new small blood vessels. Wounded animals treated with the extract had a larger percentage of dry granulation tissue, indicating a higher collagen content. Noni extract contains a variety of phytochemicals, one of which may promote collagen formation during the wound-healing process's proliferative phase. An easily available Noni juice brand, Divine Noni, was employed in this research (Noni Biotech, Chennai, India).

Human periodontal ligament cells' osteogenic differentiation and matrix mineralization were studied by Boonanantanasarn et al. [6] who used an aqueous extract of *Morinda citrifolia* leaves. Perhaps the phytochemical components of Noni leaves have a function in stimulating osteogenic differentiation and matrix mineralization. Vitamin C, triterpenes, and flavonoids were identified as active components in Noni leaves that stimulated bone formation by increasing proliferation, protein synthesis, and alkaline phosphatase activity through TGF-41. Bone marrow-derived mesenchymal stem cells have been found to benefit from flavonoids' ability to induce their differentiation into bone-forming osteoblasts. As a result, studies on animals and humans have demonstrated that *Morinda citrifolia *leaf extract has positive effects on bone and periodontal tissue regeneration, suggesting that it may be a useful osteoinductive agent.

Antimicrobial, antioxidant, anti-inflammatory, analgesic, antipyretic, anti-allergic, immunomodulatory, and anti-coagulant are only some of the benefits of *Ocimum sanctum*. These phenolic components have also been discovered because of their antioxidant and anti-inflammatory properties. Tulsi speeds up healing by boosting collagen production, and it does this in part because of the flavonoids and antioxidants it contains [13]. We tested the efficacy of an ethanol extract of *O. sanctum *L. leaves on both healthy wound healing and healing slowed by dexamethasone. The breaking strength of granulation tissue was increased significantly, as was its weight when wet or dry, and wound epithelialization and contraction occurred swiftly. Dexamethasone's anti-healing actions were similarly dramatically reduced by the extract across all wound healing modalities [14, 15].

It has been theorized that the qualities of *Morinda citrifolia *and *Ocimum sanctum *make them suitable for use as a medium in which to store avulsed teeth. The essential dry period before PDL cells are damaged beyond repair has been the subject of much research. In their study, Mahal et al. stated that the replantation success rate was greater for teeth that were returned to the mouth within 30 minutes than for those that were out of the mouth for longer. Considering that many PDL cells were destroyed but some cells survived for examination [4], a drying duration of 30 minutes was selected for the current investigation. To facilitate comparison with prior studies, the teeth in the experimental groups were kept in their designated solutions for 45 minutes.

The human premolars used to harvest the periodontal fibroblast cells were donated by healthy people needing them for orthodontic treatment. As fibroblasts account for over 60% of the PDL's cell population, they were the primary cells of interest in this study. Normal turnover, repair and regeneration, development, structure, and function of the periodontium are all aided by the PDL fibroblast. Lip, gingival, and dermal fibroblasts have some morphological similarities with PDL fibroblasts but do not function similarly in culture. PDL cells had considerably greater alkaline phosphatase and protein synthesis rates [4]. Based on these findings, we conducted an MTT test on a human PDL fibroblast cell culture model.

The purpose of this study was to use the MTT assay to investigate the impact of Noni juice and aqueous Tulsi extract on human PDL cell viability and to establish the appropriate dosage of Noni juice and Tulsi extract for future investigations. To begin with, 3-(4,5-Dimethylthiazol-2-yl)-2,5- diphenyltetrazolium bromide(MTT) is a tetrazole that is yellow in appearance but becomes purple after being digested by the mitochondria of live cells. Spectrophotometric analysis determines the concentration of the resultant purple solution. Noni juice (percentage of viable cells 10%, 5%), Tulsi (2.50%), and Tulsi aqueous extract (1.25%) concentrations were tested on PDL cells for 24 hours. According to Figure 1, only a combination of 5% Noni juice and 5% Tulsi extract was able to stimulate cell growth. Noni juice and Tulsi aqueous extract at a concentration of 5% (w/v) were shown to be most effective in stimulating the growth of human PDL cells within this range. This is why aqueous Tulsi extract and Noni juice at 5% concentration have been shown to be the best storage mediums for extracted teeth. For a concentration of Noni leaf extract lower than 0.025%, Boonanantanasarn et al. [6], found no impact on human PDL cells. A 7.5% Noni concentration, on the other hand, was fatal to the cells. The MTT assay is not without its caveats, which are affected by (1) the physiological status of the cells and (2) differences in mitochondrial dehydrogenase activity between cell types [16].

In the current investigation, grade II collagenase and dispase were used to treat the root surface, following the protocol published by Pileggi et al. [17] to protect the cells as much as possible from active trypsin. The study's positive controls showed that the method allowed for fast cell recovery and maintained cell integrity. The trypan blue dye exclusion test has been proven to be the most sensitive of the several assays. This method was selected because it can identify viable cells from non-viable ones quickly, cheaply, and easily. This is because it is possible to see, with the naked eye, the breakdown of membranes that ultimately results in cell death. Instead of soaking up the dye, healthy cells reject it, whereas non-viable cells, which have a broken membrane, absorb it. Because of this, we regarded only the cells that managed to keep the dye out to be healthy [4]. In order to determine whether or not cells are still alive, a hemocytometer and the trypan blue dye exclusion technique are often utilized. Yet this method cannot be used to assess whether or not cells are healthy and able to divide.

Compared to Tulsi, Noni exhibits significant differences in terms of the study's outcomes. Comparing Noni (percentage of viable cells 85.18%) to HBSS (84.30%) and Tulsi (68.04%), Noni had a higher mean number of viable cells. Noni has the potential to replace HBSS as a medium for storing avulsed teeth. As Noni shows promise as a storage medium, we can rule out the alternatives.

The current research design is subject to the same limitations and unpredictability as any in vitro investigation. It has been suggested in prior research [4, 17] that removed teeth may be used to replicate avulsed teeth. Perhaps extraction-related stress accounts for the observed variation in PDL cell viability counts in this investigation. The drying and breakdown of cellular metabolite is only one of the issues that these methods try to solve in replanting an avulsed tooth successfully. But, other critical factors, including pollution and the regeneration potential of the remaining fibroblasts, must also be taken into account. To prevent the avulsed tooth from becoming infected, it may be helpful to apply an antibiotic ingredient. Having a strategy for reducing absorption might be helpful as well. Determining pH and osmolality with respect to Tulsi and Noni is also one of the prerequisites before implying them as storage media in a clinical scenario. Further research needs to be done to apply the results of the present study to regular clinical use.

## Conclusions

Within the parameters of this study, we can conclude that *Morinda citrifolia *juice maintained PDL cell viability similar to HBSS. Therefore, *Morinda citrifolia *juice is an acceptable replacement for HBSS in the storage of avulsed teeth due to its low cost and ready availability.
